# ‘He’s out of control, I’m out of control, it’s just – I’ve got to do something’: a narrative inquiry of child to parent violence

**DOI:** 10.1007/s10560-022-00870-4

**Published:** 2022-08-17

**Authors:** Chye Toole-Anstey, Michelle L Townsend, Lynne Keevers

**Affiliations:** 1 School of Health and Society, University of Wollongong, Northfields Avenue, 2522 Wollongong, NSW Australia; 2 School of Psychology, Illawarra Health and Medical Research Institute, University of Wollongong, 2522 Wollongong, NSW Australia

**Keywords:** Narratives, Child-to-parent violence, Adolescent family violence, Discourse, Social work

## Abstract

Families globally experience child to parent violence (CPV). Stories of CPV have been considered at an individual and collective level to ascertain themes in parents’ accounts to identify enabling and restraining factors for CPV. However, understanding the societal narratives, defined as discourses, which have a multi-directional and entangled relationship with individual recounts of CPV have yet to be investigated. This research utilizes Narrative Inquiry with participatory approaches to explicate the societal narratives within mothers’ recounts of CPV. This analysis, guided by the interactional and discursive view of violence, and response-based practice, identifies societal narratives which set the conditions for what is possible and impossible in relation to CPV. The analysis contributes to understanding the attitudes of minimization and concealment of violence within parents’ accounts of CPV. The mothers’ recounts were constrained and made possible by the ‘good’ mother narrative and narratives of adolescence and gender. This research examines the intra-actions mothers’ recounts have with the societal narratives, and the performance of their roles as (en)actors of the subject positions ‘mother’ and ‘child’. Implications for practice and research include: consideration to practitioner’s views of power and subject positions in a parent and child relationship when working with CPV; and practitioners to be critical of essentialism and gender in working with CPV. This study posits a practical demonstration for using the response-based practice approach in research; and a way of viewing stories which can be incorporated in working with families experiencing CPV.

## Introduction

Child to parent violence (CPV) is a complicated issue experienced by families globally (e.g. Holt 2016a). CPV, also referred to as adolescent family violence, has contested conceptions (Burck et al., 2019; Coogan, 2014) and for this paper, is defined as “…a pattern of behavior that uses verbal, financial, physical or emotional mean to practice power and control over a parent” (Holt, 2013, p.2). Prevalence rates of CPV are difficult to determine, however a review by Simmons et al., (2018) found estimates of physical violence by adolescents in the community to be between 5% and 21%. Current research of CPV asserts etiological influences on a child’s use of violence (e.g. Simmons et al., 2018). Parenting has been a focus in considering enablers of CPV (Arias-Rivera et al., 2021) as well as exposure to family violence (Gallego et al., 2019). Identified impacts of CPV on parents include stress (Cottrell & Monk, 2004), isolation (Jackson, 2003), and negatively impacting peer and family relationships (Holt, 2016a, 2016b). CPV is responded to in a range of fields (Holt & Retford, 2013) including social work. Practitioners, such as social workers, identify a lack of training and differing confidence and knowledge levels as barriers to responding to CPV (Wilcox 2012; Williams et al., 2017). There is limited research examining societal factors related to CPV (Simmons et al., 2018).

Individual stories (Shelton & Johnson, 2006) have been gathered from parents experiencing CPV. Mothers identify underreporting and silencing of their stories due to fear of blame (Hunter et al., 2010), self-blame (Paterson et al., 2002), and fear of further violence (Cottrell & Monk, 2004). Mothers and grandmothers also suggest their emotions, judgement from others and lack of father figure in children’s lives as factors enabling the silencing of CPV (Williams et al., 2017). Laing (2014) identified mothers feel disappointment and resentment toward their children who use violence, alongside unconditional love and protection. Mothers’ stories identify justifications for CPV including family dysfunction, the child’s personality and mental health issues (Stewart et al., 2007). Societal and cultural influences such as peer group affiliation are identified, and gender power imbalances such as the role of the father in influencing sons’ attitudes and society accepting violence as ‘typical male behaviour’ (Stewart et al., 2007). Collectively, this research highlights the complexity and significance of the phenomena of CPV and the need for further research to examine parents experiences. To date, there has been no research of collective narratives of CPV, nor is there research elucidating societal narratives and their relations with parents’ recounts of CPV.

This study seeks to address a gap in the literature by identifying societal narratives within and across parent’s recounts of CPV. This study attends to stories from parents as formed by dominant social discourses (Arribas-Ayllon & Walkerdine, 2008). This study aims to analyse mothers’ recounts of CPV collectively in relation to societal narratives, to identify constraining and possibility-making discourses. The performed roles of mother and child are also explored to identify the embodiment of the societal narratives as well as counter-narratives to these. This paper addresses the following questions: *What societal narratives feature in a collective reading of parents’ personal recounts of CPV? What conditions of possibility and impossibility do societal narratives enable in relation to CPV?*

This paper begins by identifying the theoretical framework for the study, exploring the view of ‘narratives’ adopted and underpinnings for the research. The paper then outlines the research methods, followed by the findings, discussion and implications for practice, research and education.

## Theoretical framework

This research is situated in a storied world where narratives are “present at all times, in all places, in all societies…” (Barthes, 1975, p.237). An emancipationist paradigm (Lather, 2006) and post-positivism underpins this research. This framework positions the process of research as contributing to change, rather than only the products of the research. The lead author is a mother and sits within the world she is seeking to understand.

This study identifies narratives in two ways. First, narratives are defined as a recount of an event, with a beginning, middle and end (Labov, 1997; Wells, 2011), and in this paper are used to describe the co-constructed stories of parents. Throughout this paper, these will be called *recounts*. The second concept of narrative is the societal, institutional and cultural narratives threaded through a story, which herein are called *societal narratives*. Societal narratives are representations which epitomise broader attitudes and knowledges (Andrews et al., 2008). This second concept of narrative is aligned with discourses, where discourses are “an inherent and inseparable part of the social world, of the broader social context” (Souto-Manning, 2014, p.159). Societal discourses are not fixed, rather changing with context and time and influenced by the people who enact them, constructing and re-constructing (Arribas-Ayllon & Walkerdine, 2008). Further, discourses define what is able to be thought and felt, a way of being and speaking, in turn enabling and restraining stories (Wells, 2011). In this paper, the recounts of parents have a multi-directional relationship with societal narratives. Narratives are seen as both a way of shaping a parent’s identity and subject and agency position(ing)s (Willig, 2003), as well as influenced by and influencing societal narratives (Arribas-Ayllon & Walkerdine, 2008).

This research adopts the neologism of ‘intra-action’ (Barad, 2007) to define the relationship between societal, institutional and cultural narratives within the story of an individual. Barad (2007) introduced ‘intra-action’ in place of interaction, to stress the world as an entanglement rather than viewing both human and other-than human actors as separate. Barad’s (2007) intra-action suggests parent, child, pets and other family members ‘become with’ (Haraway 2008) each other and thereby co-emerge through the entangled relations. In relation to CPV, intra-action positions agency for the violence as entangled in the relations between actors, parent and child, in their intra-actions rather than in people and things. This study asserts recounts and societal narratives intra-act (Barad, 2007); in this way narratives are (re)produced by and modify current discourses.

### Response-based practice and violence

Response-based practice identifies concealed practices, which Jenkins (2009) asserts may abdicate responsibility for the person using violence. People enact practices classified as complicit or resistant to violence through ongoing relations, in a framework Jenkins (2009) defines as ‘invitational practice’ (Jenkins, 2009). Coates and Wade (2004, 2007) utilise response-based practice with violence, which can be applied to CPV and social work practice (e.g. Andrea Merriam et al., 2019). Coates & Wade (2007) argue misrepresentations are commonplace in narratives of violence, and at times this is deliberate as a means for victims to escape violence, reduce the severity of the violence and keep aspects of the event(s) hidden. The interactional and discursive view of violence and resistance, developed by Coates & Wade (2004) identifies four discursive operations of language about violence, that is to “(i) conceal violence (ii) mitigate perpetrators’ responsibility (iii) conceal victims’ resistance and (iv) blame or pathologise victims”(Coates & Wade, 2004, p.503).

Response-based practice is used in working with people who have experienced violence to acknowledge their resistance to the violence (Richardson & Bonnah, 2015). A response-based approach enables stories of resistance, counter-stories, to be made visible within stories of violence. Accordingly, rather than viewing accounts as blameworthy for the ‘victim/survivor’ or pathologizing the person using violence, a more holistic and generative view of social contexts beyond the individuals which support environments of violence are identified (Richardson & Bonnah, 2015). Social workers use a response-based approach to extend a ‘person in environment’ framing, enabling more supportive responses to violence (Andrea Merriam et al., 2019). Utilising response-based practice in considering stories of parents experiencing CPV, allows for socio-material conditions which make possible or impossible the violence and stories of such to be considered. The resistance of mothers to CPV is positioned as ever-present through this lens.

## Method

A Narrative Inquiry methodology (Wells, 2011) was employed for this research, incorporating participatory approaches (Reason, 2006). This approach is designed to be a tool for participants to explore power and language in shaping their stories of violence (Gaventa & Cornwall, 2001). The participatory approaches informed the interview processes, drawing from therapeutic work, to enhance parents capacity to author their own story (White & Epston, 1990).

### Recruitment and ethics

Parents were approached through organizations. Recruiting through organizations, similar to other CPV research (e.g. Laing 2014; Oviedo, 2019), aimed to support the safety of parents. If issues arose in the interview, parents were then offered to engage with a known worker or other services as appropriate. The upper age limit of 18 was applied to the child using violence, and parents were able to participate if their child was under the age of 18 at the time of using violence. No definition was provided for ‘child to parent violence’, rather parents self-selected and their definition of CPV was explored in the interview. Parents were offered a choice of the pseudonym for the research. The location of the interview was selected by parents and a retail voucher was provided for participation. The study has ethical approval from the University of Wollongong Human Research Ethics Committee.

### Data collection

Semi-structured narrative interviews were conducted face-to-face, over the phone and on Zoom. Interviews ranged in length from 40 to 95 min, with an average time of 63 min. Interviews were recorded and transcribed verbatim. Transcripts and constructed recounts were sent to parents for checking with additional questions to support iterative data analysis.

### Data analysis

This study analyses a parent’s specific recount of CPV, rather than across the whole interview, and as such Labov’s framework was employed to create a “discrete unit of discourse” to analyse (Riessman & Quinney, 2005, p.394). Recounts of CPV were constructed by the lead author using Labovs Framework as presented by Riessman (1993) to provide bounds for the analysis and checked by parents. Parent recounts of CPV were extracted from the transcript with researchers voice removed and reordered to be chronological as follows:

1. Abstract: what is the story about?
2. Orientation: who, when, where?
3. Complicating action: then what happened?
4. Resolving action.
5. Evaluation: So what? (Patterson, 2018)

This process enabled multiple recounts to be collected from interviews, with one parent contributing three recounts, two parents contributing two and remaining parents contributing one each. These recounts were analysed, rather than the transcript in its entirety. The overall transcripts were considered when analysing the narrative to account for the construction and context of the stories (Wells, 2011).

**Fig. 1:**
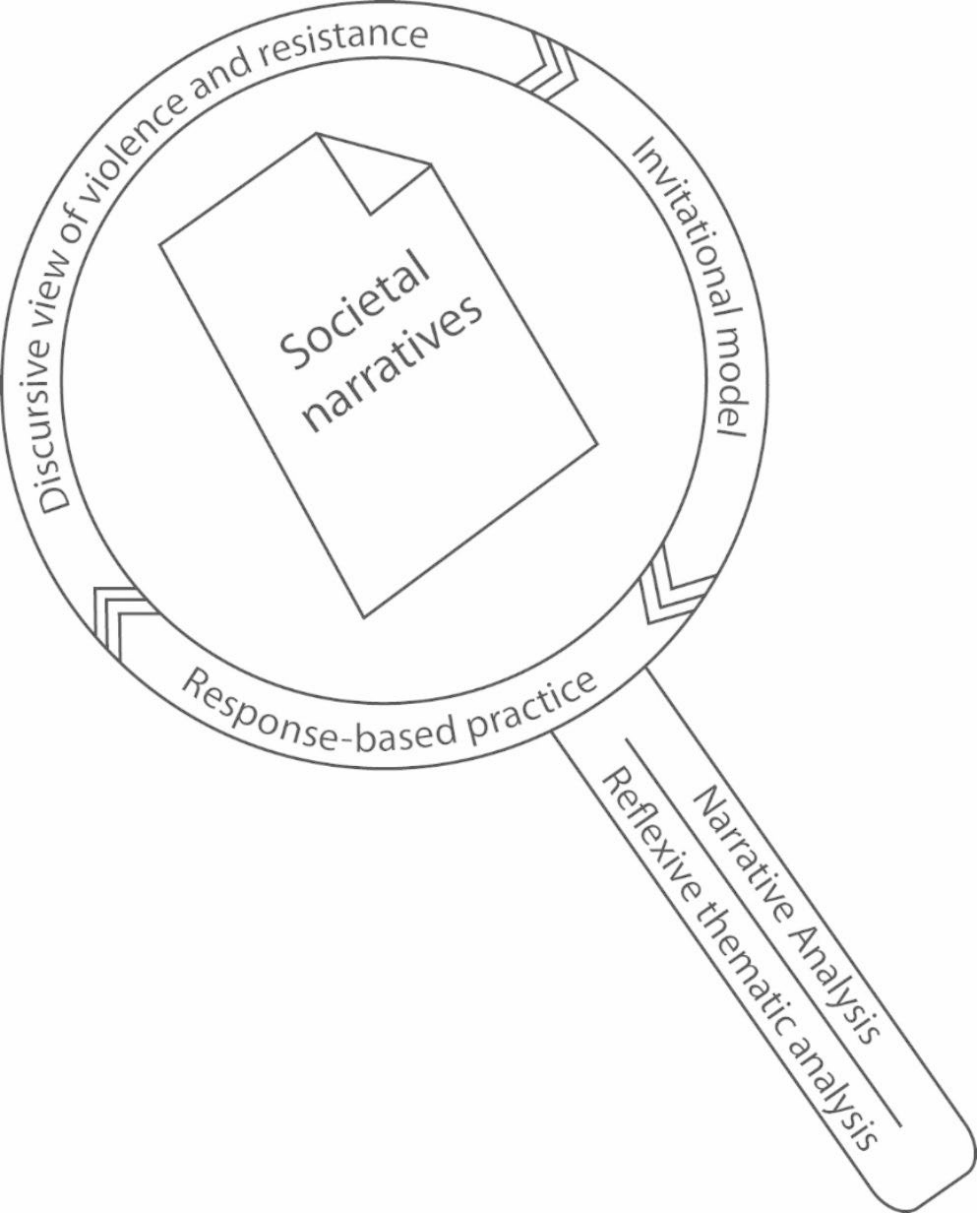
Data analysis

Reflexive thematic analysis was used to interpret the content of the recounts (Braun & Clarke, 2019; Forchtner, 2021; Riessman, 2008). Recounts of violence were closely read and reread individually, for both content and structure (Riessman, 1993): the familiarisation stage of reflexive thematic analysis (Braun & Clarke, 2014, 2019). Next, coding occurred with the following sensitizing concepts utilized to guide coding: discursive view of violence and resistance (Coates & Wade, 2007); invitational practice (Jenkins 1990, 2009) and response-based practice (Richardson & Bonnah, 2015). These sensitizing concepts also enabled an analysis of power relations in CPV. Identifying patterns across each story was then employed as the next stage, where codes were grouped into themes, and iteratively worked and re-worked. Reflexivity was applied throughout these processes including discussions between the authors (Braun & Clarke, 2021; Bruner, 1991; Wells, 2011). This process is illustrated in Fig. 1, with sensitising concepts framing the lens, while narrative and reflexive thematic analysis provide the handle for ‘analysing and writing’ (Riessman, 1993) the findings.

## Findings

Parents (n = 11) were interviewed from regional and metropolitan Australia, recruited through five organisations including homelessness, family and women’s services as well as private practitioners. Children using violence ranged from eight to eighteen years of age. Although parents were invited, all those who participated identified as mothers. Seven of the women were single parents, while others were married or partnered, although not all partners played a role in child-rearing. Some children had no siblings while others were from larger families with up to five siblings. Both male (n = 8) and female children (n = 6) enacted violence, with one family reporting two males using violence, and another family reporting multiple children using violence. Eight of the mothers experienced violence from ex-partners. In total, fifteen recounts of violence from their child were collected from mothers. Of these, one mother recounted a story of violence towards the child’s father, while fourteen were directed towards the mother.

The findings are organised into three societal narratives: the mother narrative; the adolescent narrative; and narratives of gender. These societal narratives intra-act with and within a mother’s recount, creating the conditions for what is possible and impossible in relation to CPV. As this is a study of societal narratives, the findings include literature to explicate the intra-action of the mothers’ recounts with societal narratives (e.g. Corvellec & Hultman 2012; Gordon & Paci, 1997; Riessman, 2003, 2005; Wamucii, 2011).

### The mother narrative

The societal narratives of mothering sets the conditions for the role of mothers in recounts of CPV. Positioning the mother’s role as nurturing and loving (Hill, 2019; Karr-Morse & Wiley, 2013) is part of this societal narrative. As a demonstration of nurturing, mothers stated wanting to ‘*reach*’ their child and provide care to their child due to feelings of concern. In recounts this reaching out seemed a supportive gesture, one which was often talking to the child, and was at times met with violence. One mother, Linda, described wanting to reach out to her daughter as she was concerned because her daughter previously had run away from home,

*“’Look, you know, I’m worried about you. What the hell is going on? Talk to me. Why are you doing this?' I hopped into her bed and waited for her. And she reefed me out of her bed, like pulled me from my clothes to – and just sort of tossed me on the ground”.*

Mothers’ recounts of CPV were marked by the need to maintain an ‘image’ of being a ‘good mother’ as part of this societal narrative. On a bike ride with her son Laura details, through a conversation with her child, the impossibilities created by the need to maintain the image of a ‘good mother’,

*“But yep after we talked about the situation, I just said, like, people saw, like ‘I feel like I can’t go for a bike ride again because what if the people driving part or walking saw us again? They would think ‘Oh no, those people, what happened? I said ‘The people driving past might not have thought we were mum and son, like they could have thought something completely different, because I was crying and you were running off’”.*

This recount demonstrates an act of minimising the violence, where to maintain the image of a ‘good mother’ the violence is not acknowledged at the time. It illustrates the importance of performing ‘good mothering’ (Katz, 2018), which in this instance did not include a public display of resisting violence rather a public appearance of inaction was adopted. The condition of ‘good mother’ is bi-directional in the recounts and narratives, where mothers act as good mothers to support the narrative and in turn the narrative restrains mothers actions to that of only those perceived as good.

Holding a mother responsible for her child (Coll et al., 1998; O’Brien Hallstein, 2017; Wearing, 1984) is a part of the societal narrative of mothering. This responsibility is made and remade in this societal narrative. This responsibility of mothers included ‘fixing’ the concerning issue (O’Brien Hallstein, 2017), and was a theme throughout recounts of violence. Clare illustrates this responsibility, where she tries to intervene in her son’s violence when her therapist had told her to remove herself from the situation due to complexities and danger of the situation,

*“I can’t intervene at this stuff. Through all my therapy, my plan is to warn and do all of those steps and then actually remove myself and my daughter from it if they won’t listen*”.

Here Clare, with the support of a therapist, creates the possibility of a counter-story to include alternate actions such as leaving as being within the bounds of a nurturing mother. The mother still acts to address the issues by saying *‘do[ing] all of those steps’* illustrating her nurturing and responsibility for the child, while the act of removing herself is rendered possible in her counter-story.

Self blame, a condition made possible by the societal narrative of mother, extends to self-blame for their child’s use of violence. One aspect in mothers’ self blame was the trauma a child experienced in their childhood, and was viewed as contributory to the violence. Laura demonstrates this self-blame,

*“But then I know that with the program, they were talking about trauma and stuff like that, I did have quite a bad birth experience with Matthew so I feel like his way of entering into the world and the separation of me not being with his dad and losing my dad, I feel like that’s trauma… just like… Because he’s – how do I explain it? That’s his way of emotions is to let it out and just be angry, I guess. I hope that makes sense”*.

When a society holds parents responsible for their child’s actions (Karr-Morse & Wiley, 2013; Wearing, 1984), this also includes behaviours such as violence when directed towards them. In this recount the mother felt she was to blame and culpable for the child’s trauma, and loss of the father, and in turn she is responsible for fixing this. The concept of ‘neuroculture’ in parenting (Lowe et al., 2015) is present in this societal narrative, where parents are made responsible to develop the child’s brain ‘for good or bad’ (p.199).

The recounts of CPV are examples of the multi-direction of societal narratives, where a mother blames themselves and this in turn continues to constitute the narrative mothers are responsible for CPV. Mothers’ positioned the violence as their fault, as Linda stated, *“I just felt guilty, like it was my fault that she did that”*. Mother guilt is documented in other areas of a child’s life (e.g. Seagram & Daniluk, 2002). Mother guilt is present in recounts, where Linda likens her actions the day following the violence to when mothers feel blameworthy about missing something such as a childs sporting event,

*“I think it’s going out and buying your kids something, if you’ve missed one of their sporting events. You know you try and compensate you know your belief that you’ve let them down or failure or whatever by purchasing them something you know to say, ‘Look. See, I do love you’”*.

The structure of the talk by Linda, including the use of *“you know”*, illustrates the mitigating of the child’s responsibility for their actions; that even when a child is violent towards her and she carries physical injuries, she feels guilt and blame for her daughter enacting violence. In this recount it is impossible to dis-entangle the societal narrative of mothering from the responsibility of a child for their actions which rendered the mother feeling guilt. The binding of the societal narrative of mothering to the individual recount, engenders the conditions for guilt.

The resistance to the societal narrative of mothering was also demonstrated in the mothers’ recounts, forming a counter story. One act of resistance was that children no longer occupied the subject position of ‘child’ during an enactment of violence. Some mothers, such as Laura, objectified their child as “*into the zone of the anger”* while Clare said *“this thing would descend upon him and he’d turn into this raging monster”*. This shift indicates a mother has resisted through a disconnection from the childs’ subject position, and the child instead becomes objectified in the enactment of violence with no connection to her as the mother, as Laura further explains,

“*But in that moment, when I looked at him, he wasn’t my child. He looked like some… something ese, he like just kind of – how can I say it? Like not possessed, but I felt like it wasn’t him. Does that make sense?”*

When objectifying, mothers used the structure of language to ask if that made sense, or checked in with the floating signifier ‘you know’ to see if this was understood, illustrating this counterstory needs reassurance.

### Adolescent narrative

‘Adolescence’ as a time of ‘problems’, reflecting both a developmental and societal process (Wyn & White, 1997), intra-acted with mothers recounts of CPV. This societal narrative is traced back to the industrial revolution, with mass media in the 19th century promoting the narrative of ‘juvenile delinquency’ (Savage, 2008). This narrative of adolescence was reflected in the mothers recounts as one of *‘turbulence’* which some mothers including Louise attributed to the *“intensity of hormones”*. Linda, described what she views as a ‘normal teenager’,

*“She was a normal teenage-, on her phone all the time, normal things. Having to ask her to help, all the normal stuff, but she wasn’t as aggressive and angry as then she slipped into that six to eight-month period where you know she was just uncontrollable, yelling, angry, nasty”.*

This quote demonstrates the impossibility of seeing behaviours by children at an age of adolescence as anything other than ‘adolescent problem behaviours’. Wendy extends this stating, *“you know more than the 13 year old grunts right”*. The making and remaking of the societal narrative is present here – where behaviours are labelled as problematic and this in turn continues the societal narrative of adolescents as problems.

Adolescence as a period of transition to autonomy (Ravindran et al., 2020; Wyn & White, 1997) is included in this societal narrative and seen in the mother’s recounts of CPV. Wendy, recounts this transition as a time of disconnection, creating the conditions which make it possible for a child to detach in adolescence,

*“… then he’s heading into adolescence, so now we’re talking, he’s turning about 13. He’s struggling to fit in at his high school. It was a very turbulent period and so I just feel I lost a connection with him and at the same time, he was just brewing and stewing and hiding himself away and becoming increasingly resentful and I just felt I couldn’t read him”.*

The adolescent societal narrative also constructs adolescents as ‘rebellious’ (Wyn & White, 1997, p.20). One mother conceals the violence of her daughter by dismissing some of the behaviours as typical of a rebellious adolescent. In this recount, Amy, describes her daughter finding a friend to move in with, and the conversation which ensued saying she could not move out resulted in her daughter enacting violence towards her,

*“So in June last year, my daughter decided that she didn’t wanna be at home anymore… She was being a defiant teenager basically and she had found a friend…”.*

Here the mother mitigates her daughter’s responsibility for the violence by placing it as the actions of a subject position of ‘defiant teenager’. This positioning is indicative of the multidirection of the narrative of adolescence, whereby the doing of perceived ‘deviancy’ supports a societal narrative of adolescence as problems.

The subject positioning within the narrative of adolescence can also be seen in placing some of the children who used violence as ‘at risk’, where psychological issues (Wyn & White, 1997) are incorporated in the narrative of adolescence. This societal narrative is supportive of pathologising violence, which in recounts presented as a diagnosis to address the violence. This narrative intra-acts with the mothers’ recounts, where a mother’s responsibility extends to blame for children’s psychopathology (Karr-Morse & Wiley, 2013). Amy describes the pathologising of her daughter’s childhood,

*“Yeah and I understand her childhood obviously. A lot of stuff has happened with her and she hasn’t had the most smooth-sailing childhood, but there’s definitely something there where it’s like – and I don’t believe in medication… But it took a lot to get her on medication. And to be honest, she trialled some, but it wasn’t until – two months ago that she found one that we think is actually working for her and I feel like – … I feel like I’ve got Rebecca back”*

This recount indicates the possibility of pathologising ‘at risk’ children, and while a positive experience for this mother and daughter, may minimise the responsibility of the enactions of a child to use violence. Other mothers discussed pathology as a component of their children enacting violence. Linda, associated*‘Roaccutane’* a medication for acne with the violence, while Leanne attributed the violence to her child’s *‘ADHD and the ODD’*. This pathologising, transitions a child to a subject of pathology, denying the child’s agency, thereby minimising responsibility for actions.

The possibility for a young person to be seen as a both a ‘victim’ and a ‘threat’ (Wyn & White, 1997) was included in the societal narrative of adolescence. When mothers transition their children from position of ‘child’ to a subject of pathology, the child becomes a victim where s/he is seen as a subject of their enactment of violence rather than a character of the story with agency. Transitioning the child from a ‘threat’ when using to violence to a victim of pathology in turn mitigates the child’s responsibility. This positioning also occurred when discussing violence as a learned behaviour from others. Kathryn, explained her son’s violence in relation to witnessing other male figures in his life using violence,

*“Saw his own father’s violence for that short period of time but then also saw Chris kick a door down, punch a hole in wall, shout, scream, carry on”.*

The mother presents her son as the ‘victim’ to his father’s and her partner’s violence. In this recount, the discussion of violence was made possible through the entanglement with the actions of the father. When viewing these recounts, light is given to hidden aspects of mothering, where mothers may not be as powerful and children not as powerless as dominant societal narratives make the relationship seem. These recounts provide space for a child to be positioned as both an active agent in violence, and a ‘victim’ or subject of other forces such as pathology.

### The narratives of gender

Socetial narratives of gender enable conditions of possibility and impossibility of what it is to be male and female throughout the mothers recounts. A context of patriarchy where, through interactions and relationships men have power and control over women (Radford, 2012), is the setting of this societal narrative.

In mothers recounts of CPV, daughters were situationally defined as masculine and embodying a male comic book character when enacting violence, as Linda stated,

*“And when she came in, she just flew into this rage and reefed me off the bed and threw me to the ground and, oh my god, it was just like being with the Incredible hulk, and she’s only a tiny little thing. So that’s one experience”*.

The enactment of violence, described by Linda, is characterised as inherently masculine, where to act with violence requires the body to be male. The societal narratives create the condition of impossibility for the (en)actor of violence to be female. In this recount, the child is objectified into the male comicbook character, the Incredible Hulk, in the act of violence.

The simile of the Incredible Hulk, a structure used in several mother recounts to transition their daughter from subject position of daughter to a male comicbook character, was also utilised in stories of resisting the violence. One daughter transitioned to the Incredible Hulk when supporting her mother to resist their siblings violence, as detailed by Kathryn,

*“She’s probably the one who will stand up to him and yep. I said to her ‘you looked like the hulk’, cause she’s very placid and really just chilled, and like a bit of a hippy you know. And she – just her whole demeanour changed when she did a couple of times that she has had something to say when he’s behaved that way”*.

The mother illustrates the possibility in the societal narratives of gender for her daughter to resist. In order to resist however, the daughter needed to occupy a male form. It also speaks to the female form as unable to protect itself, in need of protection through a masculine body. The constraints on a female to protect themselves was also discussed by Wendy, recounting the need for help from an outside service *“because I was a woman on my own”*. Through this statement we can see the view of women as ‘dependent’ (Hill, 2019), where although the mother socially holds a subject position of power over a child, she was dependent on a male to protect her. This narrative incorporates the role and materiality of the body in enacting and practising the embodiment of gender (Messerschmidt, 2004). The societal narratives of gender make the conditions for what is possible and impossible of our gendered bodies to enact, and these narratives indicate the constraints in viewing female’s violent enactments.

The physicality of boys and girls in the mother’s recounts of violence was viewed differently, further illustrating societal narratives of gender. In the recounts boys were seen as allowed to exhibit a certain level of violence, as Clare discussed,

*“…Another one was when one of the boys broke the other boy’s wrist and the [child protection agency] said, ‘Boys will be boys’”*.

This view minimises the violence, changing it from being a matter needing addressing to that of accepted as part of being a boy. The ‘embodied masculine presence’ (Messerschmidt, 2004) seen in this recount is illustrative of the societal narrative of gender, where it makes the conditions for possibility of acceptance of violence enacted by boys. Mothers of multiple children indicated the role of ‘rough-and-tumble play’ in children’s lives, which under other settings may also be seen as violent. Wendy details this behaviour as,

*               “They did a lot of play rumbling and wrestling and stuff like that too”*.

Play fights are sometimes seen as a way to “test out their masculinity” (Clifford-Poston, 2018, p.161) and the societal narratives of gender are supportive of boys embodying aggression. This societal narratives affects the possibility of accepting this behaviour from boys, which in turn may alter what parents may define as CPV. The intra-action of violence and male is seen in this societal narrative.

“Practicing being a man” (Clifford-Poston, 2018, p.160) can also be seen in the mothers’ recounts in relation to boys enacting what their fathers did: that was be violent towards their mothers. Kathryn recounts one of the worst thing she has done in her parenting is call her son his fathers name when he was enacting violence,

*“And I probably, in hindsight shouldn’t say what I said but I turned around, and I went ‘Okay Robert, that will be enough, and Robert is his father and he hates his father but he was just behaving like his father, and in my head, I wanted to point out to him that he was behaving like his father and that didn’t go down too well”.*

This quote illustrates how Kathryn conceptualised the son’s violence through the lens of intimate partner violence; that her experience shaped what she views as acceptable and unacceptable behaviour. Kathryn, as well as other mothers, was unable to separate the (en)actions of her child from that of her ex-partner. Narratives of gender enable the conditions for the possibility of this entanglement, where boys and fathers are grouped together as ‘males’ and the intra-action of male and violence is rendered.

Mothers recounted aspects of gendered societal narratives in relation to their parenting, entangled with the good mother narrative. A gendered view of parenting is supported by books from ‘parenting experts’ such as Steve Biddulph (Anderson & Accomando, 2002). A gendered societal narrative of parenting extends to the child in the recounts of CPV, as Wendy explores,

“*I think gender was part of the mix as well too, ‘cause the way I parented girls might have been slightly different to how I was with Samuel. I was quite familiar with how you were with girls who would be socialised a particular way and I think I had some bumpy rides with Hayley when she was about 16, 17 too but we got through that eventually, but we never kind of lost our connection through that time. I just found with Samuel, and I don’t know what that was to do with, but we lost that connecting coming around that age”*.

Wendy illustrates how the gender of her son influenced her parenting, through the structure of comparing parenting her daughters. Fathers were absent in this research and many fathers were absent from their child’s lives as recounted by their mothers. Laura explains the absence of a father in her child’s life,

"*And I feel like not having that male constantly in his life and authority sometimes, for someone else to step up, he puts that on me. So I think it’s I’m the blame for a lot of things"*

This mother takes the blame for the absence of the father. Intra-acting with the mother narrative, she feels there is something she cannot give her child because she is not a male. However, assumed in this mother’s recount is not only a deficit view of a mother in relation to authority over a child and the need for a child to have authority over them, but that a male provides something a mother cannot. This encompasses an essentialising concept (Prentice & Miller, 2006) where the narrative renders it impossible for a mother to meet the entire needs of their son.

## Discussion

This study sought to understand the societal narratives entangled with a parents recount of CPV, and the conditions of possibility and impossibility societal narratives enable in relation to CPV. The current study is the first, to the authors knowledge, to examine recounts of mothers experience of CPV with a theoretical frame of response-based practice and an interactional and discursive view of violence. Similarly, the Baradian view of intra-action is a novel application to CPV. This research demonstrates the influence of societal narratives within a parent’s recount of CPV, namely that of mothering, adolescence and gender. Significantly, these findings extend understanding of the: subject positions entanglement and arrangement in CPV; the views of gender in an act of CPV; and the role of response-based practice in research. This present study contributes to the understanding the attitudes of minimization and concealment of the violence occurs within mothers’ accounts of CPV.

This study contributes to understanding the self-blame of mothers for CPV indicated in previous studies (e.g. Brule & Eckstein 2016; Selwyn & Meakings, 2016; Williams et al., 2017) and the shame mothers experience (Miles & Condry, 2015; Omer & Lebowitz, 2016). The societal narratives within mothers’ recounts bring forth the possibility of a mother’s self-blame. This self-blame becomes with the ‘good mothering’ narrative, and also the role of the narratives in limiting the possibility of seeing the issue of CPV outside of the entangled parent and child relationship. This research further expands on previous research on the role of the absent father (Edenborough et al., 2008; Williams et al., 2017), demonstrating how this absence both mitigates a child’s responsibility for the enactment of violence and continues to essentialise gender.

This paper contributes to understandings of the intra-action of discourses on recounts of CPV. Mothers’ stories illustrate the intra-action of societal narratives with the individual recounts, which shape the way children’s actions and parent’s responses are made possible and constrained. Further, the pathological contributors to CPV have been noted (see Simmons et al., 2018) and this study progresses this research to indicate the possibility for pathology within the current societal narratives of adolescence. This study offers an alternate view to pathologising the violence, demonstrating the constraining role of the narratives to see a child’s violence as anything but a pathology, thereby minimising a child’s responsibility.

### ‘Mother’, ‘child’ and ‘cender’ entanglement

This study makes a contribution in relation to the subject positions, and transitions of, ‘mother’ and ‘child’. A Baradian view (2007) applied in this research posits these positions of ‘mother’ and ‘child’ are a product of the intra-action within particular social, political, historical and material ‘arrangements’. This paper contributes the particular ‘arrangements’ which make CPV possible in a mother and child relationship include the societal narratives of gender, mothering and adolescence. The intra-action of mother and child subject positions makes it impossible to attribute blame to either position. This intra-action does not abdicate responsibilities, as Barad’s (2007) relational ontology increases accountability and responsibility. This view highlights the subject positions of mother and child may not be as binaried as the subject positions distinguish them as, but rather these positions ‘become with’ and are entangled. Further, this paper finds the societal narratives engender the impossibility of seeing CPV outside of the parent and child. This impossibility for CPV positions the roles of mother and child as the parameters for the violence, with minimal consideration able to be applied for the ‘arrangements’ which makes it possible. The mothers’ recounts show the dislocation of mother and child relationships in the act of violence, where the societal narratives render it impossible to consider ones own child as enacting violence rather objectifying the child to that of a comic book character is more palatable.

The societal narratives of subject positions ‘mother’ and ‘child’ sets the possibilities and impossibilities for these roles as well as the parameters of interaction. Holt & Shon (2018, p.926) theorise in their research that CPV may be “a product of mundane family processes, the extent of which are shaped by an interaction between different generational (e.g. adolescental/parental) and genders (e.g. maternal/paternal) pre-occupations”. This study finds the entanglement and intra-action of the subjects ‘mother’ and ‘child’, and the oscillation of power between the two roles and gender. Societal narratives position parents as the ‘custodians of power’ (Oviedo, 2019), typically characterising the parent as ‘powerful’ and the child as ‘vulnerable’ (Holt 2013). The mothers’ recounts illustrate more complex power dynamics in a parent and child relationship, which are not dualistic but rather oscillating, multi-directional and encompassing gender. Additionally, the mothers’ recounts demonstrate complex power dynamics in parent and child relationships with CPV (Holt, 2011; Tew & Nixon, 2010). This view emphasises the intra-action above the individuals, and as contingent on the arrangement (Barad, 2007). Power in these recounts did not sit solely with the mother, nor with the child, rather similar to Holt (2013) views, power is distributed and constantly in a state of flux. Sometimes this flux was an act of mitigating the child’s responsibility, other times it was active resistance and produced a counter-story. This demonstrates the intra-action of parent, child, and gender requires ongoing enactment to maintain non-violent relations. Further, it moves the responsibility for the enactment of violence from solely that of the child or the mother, to within a context which makes these actions possible.

This paper advances societal understandings of ‘mothering’, elucidating the role of gender in parenting, and narratives which position responsibility for development of the child as a mothers’ domain. Entangled with this narrative was other narratives of gender, which essentialise the role of the father.

The individual recounts, as enabled and restrained by societal narratives, cast CPV as a two person problem, one solely between the child and parent and exacerbated by issues of gender and pathology. The narratives create the impossibility of seeing CPV beyond the family, to consider structural arrangements supportive of the violence, or an intersectional lens to illustrate the impact of class, race, education, and other factors. This study provides a contribution to difficulties experienced by mothers of viewing violence when enacted by a daughter. The entanglement of violence with masculinity is present in the recounts, and is positioned in this study as restraining the recounts of mothers.

### Response-based practice

This study provides evidence for the use of response-based practice as a theoretical framework for research. Through employing this approach within the theoretical frame, mothers recounts of CPV demonstrated structures of narratives to minimise and conceal the violence including a mother’s self-blame, essentialising absent fathers, pathologising the child and the impossibility of violence being enacted by females.

## Limitations

The findings should be interpreted in relation to the limitations of the study. First, the study has a small sample size, although is drawn from a range of locations across Australia. The sample size of the study was impacted by COVID-19. Second, the mothers interviewed are a fairly homogenous group demographically as all parents who participated were women and most experienced intimate partner violence. Third, this study focused on mothers retelling recounts of individual acts of violence and this may affect the generalizabilty of these findings.

## Implications

Based on the findings of this research, there are implications for practitioners, research and education. First, practitioners should consider a mother’s recount within the arrangement of societal narratives. This will support practitioners to understand the societal narratives which make possible a mothers’ self-blame, the pathologizing of a child’s violence and make it impossible to see violence as enacted by a child. Practitioners should consider the role of gender essentialism in recounts of CPV, including the role of the father and views of violence as inherently masculine. Practitioners should be aware of the enabling and constraining role societal narratives have on viewing CPV. Second, future research and practice with CPV could utilize the response-based practice to explicate the role of societal narratives in minimizing and concealing violence within recounts. Future research to understand the role of parents’ recounts of CPV in formulating responses to the violence is needed. Further research with larger more diverse samples may support more generalizable findings. Third, education for practitioners should include the role of societal narratives, to support practitioners to understand the influence societal narratives have when sharing and viewing experiences of violence. Throughout practice, research and education, consideration is needed to the discourses of essentialism, gender and parenting. Finally, the intra-action framework applied in this research should be used in CPV practice and research to allow for alternate viewings of power within a parent and child relationship.

## Conclusions

This study adds to understanding of the experience of CPV. The role of societal narratives, particularly in relation to the conditions of impossibility and possibility made and remade by the narratives, is demonstrated in this paper. With insights provided in the framework of response-based practice, this study illustrates the impact of societal narratives of the mothering, gender and adolescence. These results provide preliminary evidence for practitioners, including social workers, to be aware of the role of societal narratives when working with and researching CPV, as well as the acts of resistance, minimising and concealing of the violence which may occur.
